# Rapid Characterization of Tanshinone Extract Powder by Near Infrared Spectroscopy

**DOI:** 10.1155/2015/704940

**Published:** 2015-03-18

**Authors:** Gan Luo, Bing Xu, Xinyuan Shi, Jianyu Li, Shengyun Dai, Yanjiang Qiao

**Affiliations:** ^1^Beijing University of Chinese Medicine, Beijing 100029, China; ^2^The Key Laboratory of TCM Information Engineering of State Administration of Traditional Chinese Medicine, Beijing 100029, China

## Abstract

Chemical and physical quality attributes of herbal extract powders play an important role in the research and development of Chinese medicine preparations. The active pharmaceutical ingredients have a direct impact on the herbal extract's efficacy, while the physical properties of raw material affect the pharmaceutical manufacturing process and the final products' quality. In this study, tanshinone extract powders from Salvia miltiorrhiza which are widely used for the treatment of cardiovascular diseases in the clinic are taken as the research object. Both the chemical information and physical information of tanshinone extract powders are analyzed by near infrared (NIR) spectroscopy. The partial least squares (PLS) and least square support vector machine (LS-SVM) models are investigated to build the relationship between NIR spectra and reference values. PLS models performed well for the content of crytotanshinone, tanshinone IIA, the moisture, and average median particle size, while, for specific surface area and tapped density, the LS-SVM models performed better than the PLS models. Results demonstrated NIR to be a valid and fast process analytical technology tool to simultaneously determine multiple quality attributes of herbal extract powders and indicated that there existed some nonlinear relationship between NIR spectra and physical quality attributes.

## 1. Introduction

Pharmaceutical powders, described as heterogeneous systems with different chemical and physical attributes, are the main source of oral solid preparations. It is estimated that more than 80% of the drug production is based on powders in a typical pharmaceutical industry [1]. As the input of the pharmaceutical process, powders have great impacts on the whole production process. Tiny quality fluctuations of powders may result in batch to batch variations of the final products [2–4]. For example, the performance of the granule tableting process would be deteriorated significantly when the initial moisture content of microcrystalline cellulose was increased from 2.6% to 4.9%, the values of which are considered to be within normal variations of the moisture content (i.e., 3–5%) for microcrystalline cellulose. On the other hand, the flow ability of the granule was improved as the initial moisture content of microcrystalline cellulose was increased [4]. Therefore, it is necessary to understand and control the critical material attributes of powders at the beginning of pharmaceutical processes.

Process analytical technology, launched by the United States Food and Drug Administration [5], is often used to monitor and control critical quality attributes of raw materials and in-process products to ensure the quality of final products. Process analytical technology approaches, which are based on scientific knowledge and risk analysis, afford the design and development of efficiently controlled process. In this way, it is possible to realize the preset target of the product when the manufacturing process is finished. Common process analytical technology tools used in rapid evaluation of chemical and physical properties of powder are as follows: near infrared spectroscopy [6, 7], Raman spectroscopy [8], Raman chemical imaging [9], acoustic emission [10, 11], and so forth. Among them, near infrared spectroscopy (NIR) is the most widely used process analytical technology tool in the pharmaceutical process monitoring and control [12, 13], since it is fast, nondestructive, and of low-cost. Compared with the imaging technology, NIR is rapid and of low-cost. And for the analysis of complex system with many ingredients, that is, herbal materials [14, 15], NIR shows unique advantages over other spectroscopy technologies, such as Raman spectroscopy which does well in the analysis of pure compounds [16].

NIR spectra carry abundant information not only on chemical compositions but also on physical properties (e.g., particle size) of the sample [17, 18]. In powder analysis, some qualitative work with NIR has been reported, such as rapid identification of the production area [19] and brand traceability [20]. And some work about quantitative analysis has also been done in relating the NIR spectra to different quality attributes of powders, such as content of ingredients [6], flow ability [21], content of moisture [22], and particle size [23]. And subsequent unit operations of solid dosage preparations could benefit from the quality control of powders.

Herbal extract powders as raw materials play an important role in the research, development, and manufacturing of Chinese medicine preparations. Currently, the quality control of herbal extract powders mainly focuses on the content of active pharmaceutical ingredients according to the Chinese Pharmacopoeia (Ch.P.) 2010 [24]. However, like powders of chemical materials, it is far from enough to control the quality of herbal extract powders only by the content of components. In order to understand and control the quality of herbal extract powders as well as related manufacturing processes and products, physical properties of herbal extract powders should be paid more attention. Generally, classical methods for determination of physical properties of herbal extract powders were time-consuming. And, recently, NIR spectroscopy has been reported to be successfully applied in the analysis of herbal powders, where the contents of active pharmaceutical ingredients were the attractive part. Quantification of contents of two or more ingredients in herbal extract powders could be carried out by NIR [15, 25]. Nevertheless, the application of NIR to analysis of physical properties of herbal extract powders is still an untouched area. Therefore, the aim of this paper is to investigate the possibility of using NIR to predict both the chemical and physical properties of herbal extract powders at the same time. To the best of our knowledge, this is the first report on the application of NIR spectroscopy in characterization of multiple quality attributes of herbal extract powders. It is expected that the usage of NIR could be broadened in the quality control of raw materials of herbal products.

The rest part of the paper is organized as follows: firstly, contents of cryptotanshinone and tanshinone IIA of 50 batches of tanshinone extract powders were determined by high performance liquid chromatography (HPLC), and the physical quality attributes were measured by classical methods. Then, the NIR spectra of tanshinone extract powders were collected in diffuse reflection mode and different data pretreatment methods were screened. After that, partial least squares (PLS) and least square support vector machine (LS-SVM) models for quantitative prediction of different quality attributes were built, and the performances of these models were compared. Finally, a conclusion of this paper is provided.

## 2. Experimental

### 2.1. Materials

*Salvia miltiorrhiza* (batch number: 20120402) were purchased from Beijing Ben Cao Fang Yuan Pharmaceutical Co., Ltd. (Beijing, China). Cryptotanshinone (batch number: 1110852-200806) and tanshinone IIA (batch number: 110766-200619) were obtained from National Institute for the Control of Pharmaceutical and Biological Products (Beijing, China). Acetonitrile (Fisher scientific, Waltham, Massachusetts, USA), phosphoric acid (Fisher scientific, Waltham, Massachusetts, USA) and distilled water (Hangzhou Wahaha Group Co., Ltd., Hangzhou, China) were of HPLC grade and all other reagents were of analytical grade.

30 batches of alcohol extracts of* Salvia miltiorrhiza* were purchased from 5 different suppliers. 20 batches of alcohol extracts of* Salvia miltiorrhiza* were homemade under central composite experimental design in order to expand the variation coverage of the sample sets. Factors and levels of the alcohol extraction process are shown in Table 1, and the experiment schedules are listed in Table 2. The alpha value was set to 1 and the replication of center points was 5. The extraction process was carried out according to procedures specified in the Chinese Pharmacopoeia (Ch.P.) 2010 [24] as follows: pulverized powders of* Salvia miltiorrhiza* were extracted by alcohol through heating reflux, after which the alcohol was filtered and the filtrates were merged. Then, the filtrate was vacuum evaporated to recover ethanol, enriched to 1.30~1.35 of the relative density at 60°C, washed to colorless by hot water, dried at 80°C, and crushed to fine powders finally. As a result, 50 batches of alcohol extracts of* Salvia miltiorrhiza* were used in the following experiments.

## 3. Method

### 3.1. HPLC Analysis

5 grams of tanshinone extract powders was taken to a 5 mL volumetric flask after a precise weighing, dissolved by methanol, and then diluted with methanol to volume. All samples were filtered through a millipore membrane filter with an average pore diameter of 0.45 *μ*m, and 10 *μ*L filtrate was injected into the HPLC system for analysis.

The contents of cryptotanshinone and tanshinone IIA were quantitated by the reverse phase HPLC according to Ch. P. 2010 [24]. An Agilent 1100 HPLC system (Agilent Technologies, Santa Clara, California, USA) with a vacuum degasser, a quaternary pump, an autosampler, a thermostatic column compartment, and a diode array detector were used. Separation was performed on Agilent SB C_18_ column (250 mm × 4.6 mm with 5 *μ*m particle size) at 30°C. The mobile phase consisted of (A) acetonitrile and (B) 0.026% phosphoric acid water solution. The gradient elution was as follows: linear change from (A) 0 to 60% at 0–20 min and linear change from (A) 60% to 80% at 20–50 min. The signal was monitored at 270 nm. The flow rate was maintained at 0.8 mL·min^−1^. Reequilibration duration was 10 min between individual runs.

### 3.2. NIR Spectroscopy

The NIR spectra were collected in the integrating sphere mode using an Antaris Nicolet FT-NIR system (Thermo Fisher Scientific Inc., Waltham, Massachusetts, USA). About 2 grams of powders was used with compaction in each test. Each sample spectrum was a result of 64 scans in the range between 10,000 and 4000 cm^−1^ using 8 cm^−1^ resolution at ambient temperature and was recorded by log⁡1/*R* with air as reference. Every sample was scanned three times and the final spectrum was an average of the three. All NIR spectra were collected and archived using the Thermo Scientific Result software.

### 3.3. Physical Attributes Determination

The specific surface area was determined by the 3h-2000a automatic specific surface area analyzer (Beishide Instrument Technology (Beijing) Co., Ltd., Beijing, China) according to the multimolecular layer absorption theory. In reference mode, with nitrogen as the absorbate and purge medium and helium as carrier gas, the test was purged for 60 minutes at 30°C.

The particle size distribution was determined by the bt-2001 laser particle size analyzer (Dandong Bettersize Instruments Ltd., Dandong, Liaoning, China). Based on the light scattering theory, measurements were obtained using dry dispersion with air as medium and the refractive index of sample is 1.520. The *D*_10_, *D*_50_, and *D*_90_ values are calculated to represent the maximal particle size diameters that include 10%, 50%, and 90% of the particles, respectively. For example, the *D*_90_ value means that 90% of particles are smaller than this particle diameter, whereas the remaining 10% of the particles have larger diameters. Each sample was tested for three times, and the average value was taken.

The tapped density was analyzed by the hy-100 powder density tester (Dandong Hengyu Instruments Ltd., Dandong, Liaoning, China). According to the European Pharmacopoeia 8.0 [26], 5 grams of powders was poured into the measuring cylinder. Afterwards, the volumetric measurement was made following 1250 taps, which has been described as the number of taps sufficient to achieve maximum compaction equilibrium [27]. The final volume (Vt) was used to compute the tapped density. Each sample was measured in triplicate, and the average value of density was taken.

The moisture content of sample was determined by the Sartorius ma-35 moisture analyzer (Sartorius AG, Gottingen, Germany). This test needs about 2 grams of powders being heated for 10 minutes at 105°C.

### 3.4. NIR Spectra Pretreatment

A variety of preprocessing methods for the spectroscopic data were compared to extract the useful information from noise, such as normalization, baseline, Savitzky-Golay smoothing, Savitzky-Golay smoothing plus first-order derivatives, Savitzky-Golay smoothing plus second-order derivatives, spectroscopic transformation, multiplicative scatter correction, standard normal variate transformation, and wavelet de-nosing of spectra. SIMCA P +11.5 (Umetrics AB, Umea, Sweden) and Unscrambler 9.7 (Camo software, Oslo, Norway) served as chemometric tools for data preprocessing.

### 3.5. Model Building

In order to build quantitative models, the samples were split into the calibration and validation sets by Kennard-Stone algorithm. In this study, 40 samples were selected as the calibration set, while the remaining 10 samples were kept as the validation set. The whole spectra with wavenumber 10000–4000 cm^−1^ were used to build models.

PLS regression algorithm performed on Matlab version 7.0 (Mathworks Inc., Natick, Massachusetts, USA) with PLS Toolbox 2.1 (Eigenvector Research Inc., Wenatchee, Washington, USA) was used to set up quantitative models. The number of latent variables was optimized by the leave-one-out cross validation method and predicted residual error sum square (PRESS). The performances of PLS models were evaluated in terms of correlation coefficient *r* for both calibration and validation sets (*r*_cal_ and *r*_pre_, resp.), the root mean square error of calibration (RMSEC), the root mean square error of cross validation (RMSECV), the root mean square error of prediction (RMSEP), BIAS for both calibration and validation (BIAS_cal_ and BIAS_pre_, resp.), and the relative predictive deviation (RPD). PLS model showed good performance with large *r* and RPD values, while small RMSEC, RMSECV, RMSEP, and BIAS values. The equations of these indicators were as follows:

(1) $$\begin{array}{c} r=\frac{\sum_{i=1}^{n}\left(C_{i}-{\bar{C}}_{i}\right)\left(C_{pi}-{\bar{C}}_{pi}\right)}{\sqrt{\sum_{i=1}^{n}{\left(C_{i}-{\bar{C}}_{i}\right)}^{2}}\sqrt{\sum_{i=1}^{n}{\left(C_{pi}-{\bar{C}}_{pi}\right)}^{2}}},\\ \text{RMSE}=\sqrt{\frac{{\sum_{i=1}^{n}\left(C_{pi}-C_{i}\right)}^{2}}{n}},\\ \text{BIAS}=\frac{\sum_{i=1}^{n}\left|C_{pi}-C_{i}\right|}{n},\\ \text{RPD}=\frac{{\text{SD}}_{\text{pre}}}{\text{RMSEP}},\\ {\text{SD}}_{\text{pre}}=\sqrt{\frac{\sum_{i=1}^{n}{\left(C_{ip}-{\bar{C}}_{ip}\right)}^{2}}{n-1}}, \end{array}$$

where *n* is the number of samples, *C*_*i*_ is the reference value of the sample of number *i*, *C*_*pi*_ is the predictive value of the sample of number *i*, ${\bar{C}}_{i}$ is the average value of reference value, and ${\bar{C}}_{pi}$ is the average value of predictive value. SD_pre_ is the standard deviation of prediction set data, *C*_*ip*_ is the reference value of prediction set, and ${\bar{C}}_{ip}$ is the average value of prediction set.

LS-SVM algorithms carried out by LS-SVM lab Toolbox 1.8 (Department of Electrical Engineering, Leuven-Heverlee, Belgium) [28] was also used to set up quantitative models. In order to obtain the LS-SVM model, two extra hyperparameters, gam and sig^2^, need to be tuned by leave-one-out cross validation. Gam is the regularization parameter, determining the tradeoff between the training error minimization and smoothness of the estimated function. Sig^2^ is the Gaussian RBF kernel function parameter. The performance of the LS-SVM model was evaluated in terms of chemometric indicators the same as PLS regression.

## 4. Results and Discussion

### 4.1. HPLC Determination of Cryptotanshinone and Tanshinone IIA

For quantitative consideration, the calibration curves of cryptotanshinone and tanshinone IIA were established upon eleven consecutive injections of different concentrations. The concentration range is from 0.73 to 102.48 *μ*g·mL^−1^ for cryptotanshinone and from 0.83 to 182.56 *μ*g·mL^−1^ for tanshinone IIA. Regression equation calibrated for cryptotanshinone was *y* = 46.95*x* + 2.506 (*R* ^2^ = 0.9999, *n* = 11) and *y* = 44.74*x* + 19.93 (*R* ^2^ = 0.9999, *n* = 11) for tanshinone IIA. Seen in Table 3, it is obvious that contents of cryptotanshinone and tanshinone IIA varied considerably among different samples. And large quality fluctuations could be observed among the commercial tanshinone extracts produced under the same specifications according to the Ch.P. 2010 [24]. For example, contents of cryptotanshinone in commercial extract powders varied from 2.8 to 88 mg·g^−1^, with the mean value of 12 mg·g^−1^ and the standard deviation of 28 mg·g^−1^. And, for tanshinone IIA in commercial extract powders, the contents were from 0.85 to 1.4 × 10^2^ mg·g^−1^ with the average value and standard deviation being 25 and 27 mg·g^−1^, respectively. The possible reasons could be attributed to different sources of* Salvia miltiorrhiza*, different preparation processes, and various storage conditions.

### 4.2. Analysis of Physical Attributes

The results of physical attributes tests are shown in Table 4. The relative standard deviations of all physical attributes were smaller than 0.5. It is clear that the variation coverage of physical properties were smaller than the contents of active pharmaceutical ingredients, whose relative standard deviations values were above than 1.0.

Generally, the specific surface area of loose porous material is supposed to be large due to plenty of micropores. But values of specific surface area for the homemade and commercial* Salvia miltiorrhiza* extract powders were all below 0.500 m^2^·g^−1^, indicating that these samples were dense with little micropores. If such extract powder was used as raw material for dry granulation or direct tableting, the dense structure might result negatively in the dissolution tests of produced granules or tablets.

Homemade extract powders were treated by grinding, while commercial extract powder was directly from spray drying. The two different preparation methods may lead to the variation of particle size distribution between the two sample sets. *D*_50_ values were 15.34~57.17 *μ*m for commercial samples and those were 35.52~83.33 *μ*m for homemade samples, suggesting that spray drying powders were finer than grinding ones. In real pharmaceutical applications, that is, granulation or tableting, fine powders made from spray drying as raw materials are a better choice than coarse powder, since fine powders deserve better uniformity of distribution.

The tapped density values of homemade samples are close to that of commercial samples. Different from the liquid density, solid density is not a unique “band.” Tapped density of herbal extract powders with different chemical compositions and contents may be the same. For example, as seen in Table 5, contents of cryptotanshinone for the first three samples are 3.4, 37, and 33 mg·g^−1^, and contents of tanshinone IIA are 1.5, 2.5, and 28 mg·g^−1^, respectively. The three samples had the same tapped density 0.74 g·cm^−3^, while the former two samples had similar contents of tanshinone IIA, and the latter two samples had similar contents of cryptotanshinone. In contrast, tapped density of the extract powders with similar chemical compositions may be different. The contents of cryptotanshinone of the last two samples in Table 5 are 4.0 and 4.3 mg·g^−1^, and the contents of tanshinone IIA of them are 2.0 and 2.9 mg·g^−1^, indicating that the chemical compositions and contents of these two samples are similar, but the tapped densities are 0.77 and 0.70 g·cm^−3^, respectively.

Similar to tapped density values, values of moisture content did not show much difference. Most of moisture contents were below 5% except for two samples of homemade sample sets. Moisture of extract powders could directly affect the subsequent operations, such as dry granulation and direct compaction. If the moisture content is too high, the storage of extract powders will be a challenge. While, if the moisture content is too low, it will be difficult for direct compaction of tablet. So moisture of tanshinone extract powder should be monitored and controlled within a proper range.

It could be summarized that, for each quality attribute, the values fluctuated within a certain range. The differences of tapped density (the values of relative standard deviation being 0.083 for homemade and 0.096 for commercial) were smaller than other indexes (all values of relative standard deviation being more than 0.15 for the homemade and commercial). Tapped density is macroscopic, while other quality attributes are microscopic, which may lead to the different variation coverage of measured indexes.

### 4.3. Data Pretreatment

**Figure 1:**
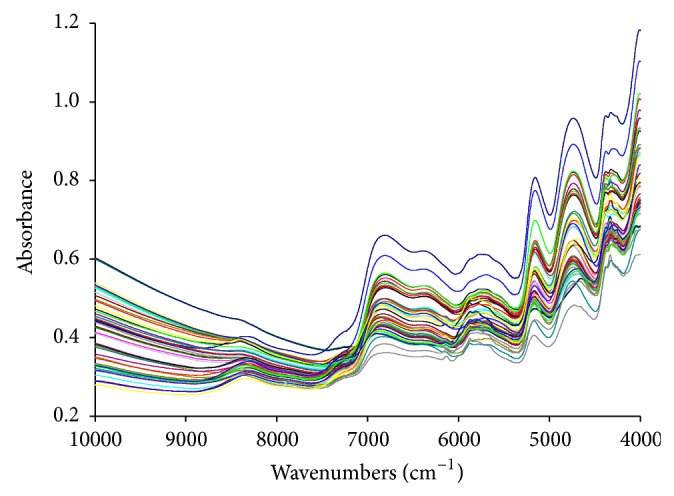
The near infrared spectra of 50 samples without any pretreatment.

Figure 1 shows the raw NIR spectra without any pretreatment. In the region of wavelength 7000~4000 cm^−1^, serious peak overlapping and great noise could be observed, suggesting that a great deal of information may be concealed.

**Figure 2:**
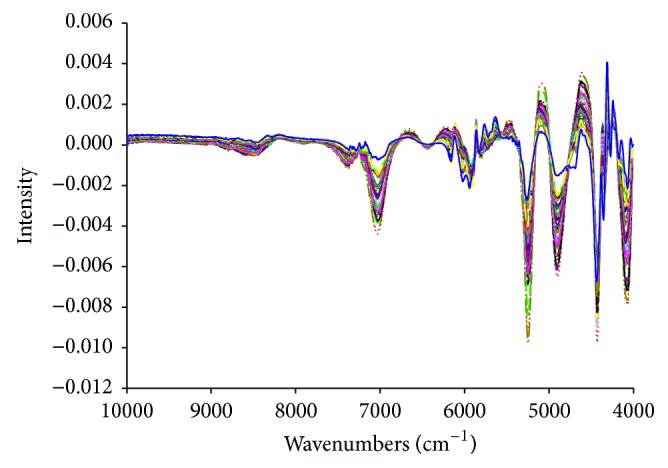
The near infrared spectra of 50 samples after Savitzky-Golay smoothing plus first-order derivatives.

For different quality attributes, the NIR spectra with different data pretreatment methods were found to bear different capability in both calibration and validation. As shown in Tables 6 and 7, the best preprocessing methods in prediction of the contents of cryptotanshinone and tanshinone IIA are normalization and Savitzky-Golay smoothing plus first-order derivatives, respectively. Normalization could eliminate redundant information and increase the difference among samples. Savitzky-Golay smoothing could clear high frequency noise by means of least square polynomial fitting to the data in the moving window. And 1st derivative spectrum could eliminate shift irrelevant to the wavelength. As shown in Table 8, the best preprocessing method for specific surface area, *D*_10_, *D*_50_, and moisture content is Savitzky-Golay smoothing plus first-order derivatives.  Figure 2 shows the NIR spectra after Savitzky-Golay smoothing plus first-order derivatives, where the shifted baselines of the raw spectra are corrected. For *D*_90_ and tapped density, the best preprocessing methods are spectroscopic transformation and wavelet denoising, respectively. Spectroscopic transformation is often used to switch between absorbance and reflectance data and transform reflectance data into Kubelka-Munk units. And wavelet denoising deals with high frequency noise of spectrum.

### 4.4. Calibration and Validation of Quantitative Models

**Figure 3:**
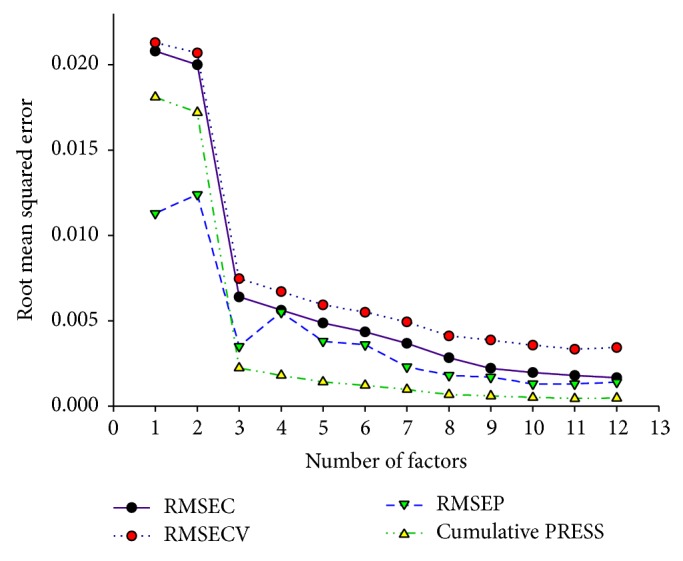
Calibration characteristics versus number of latent factors for the content of cryptotanshinone. RMSEC, RMSECV, and RMSEP represent the root mean square error for calibration, cross validation, and prediction, respectively. PRESS means predicted residual error sum square.

The calibration results of the content of cryptotanshinone (see Figure 3) demonstrate that 11 latent factors with minimum RMSECV and PRESS values are enough to build the PLS models. The correlation coefficients of calibration and validation sets were 0.9963 and 0.9969, respectively. The values of root mean square error for calibration, cross validation, and prediction were 0.0018, 0.0033, and 0.0013 mg·g^−1^, respectively. And the RPD value was 8.9.

**Figure 4:**
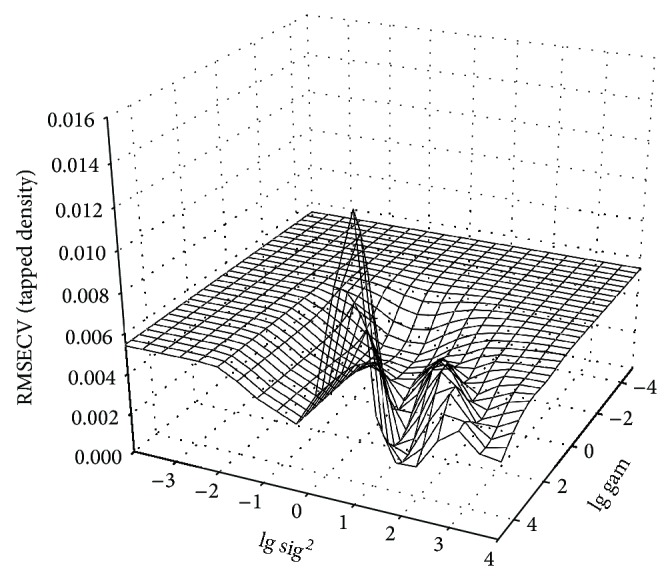
Hyperparameters optimization of the least squares support vector machine model for tapped density. RMSECV represents the root mean square error of cross validation. Gam is the regularization parameter, and sig^2^ is the Gaussian RBF kernel function parameter.

Gam and sig^2^ of the LSSVM model for the tapped density are optimized by the standard simplex algorithm and resulted values are 6.9355 and 111.63, respectively (see Figure 4). The correlation coefficients of calibration and validation sets were 0.9851 and 0.8875, respectively. The values of root mean square error of prediction were 0.020 g·cm^−3^. And the RPD value was 2.2.

Furthermore, the established PLS and LS-SVM models are compared, as shown in Tables 8 and 9. It can be found that PLS models exhibited good performance in prediction of chemical properties, particle size, and moisture content. But, for specific surface area and tapped density, LS-SVM models performed better than PLS models. Take the tapped density for example, the correlation coefficients of calibration and validation sets for the PLS model were 0.8830 and 0.8940, respectively. The root mean square error for calibration, cross validation, and prediction were 0.034, 0.038, and 0.023 g·cm^−3^, respectively. The RPD value was only 1.9. In contrast, the correlation coefficients of calibration and validation sets for the LS-SVM model were 0.9851 and 0.8875, respectively. The root mean square error of prediction was decreased to 0.020 g·cm^−3^. And the RPD value was increased to 2.2.

For all quality attributes, the performances of LS-SVM models were slightly better than PLS models. As stated in [29], the LS-SVM models could take into account some nonlinearity between the dependent and independent variables, while improved PLS models with the low prediction abilities. That is to say, there may be some nonlinear relationship between the NIR spectra and quality attributes. However, in prediction of the content of cryptotanshinone and tanshinone IIA, particle size, and moisture, PLS models were sufficient, since they are easy to be implemented. While, for physical attributes, such as the specific surface area and tapped density, where the prediction of PLS models did not perform well, LS-SVM model may be a better choice.

## 5. Conclusions

In this paper, the chemical and physical quality attributes of tanshinone extract powders are determined simultaneously by near infrared spectroscopy for the first time. The PLS and LS-SVM models are used to build quantitative models. It is found that PLS models exhibit good performance in prediction of the chemical properties, particle size (*D*_10_, *D*_50_, and *D*_90_), and moisture content. And the LS-SVM models are good at predicting the specific surface area and tapped density. Results demonstrated that the massive information concealed in NIR spectra could be analyzed with the help of a combination of process analytical technology tools and chemometric methods. The subsequent process operations, such as blending, granulation, and tableting and even the final products could benefit from the understanding and control of herbal extract powders.

## Figures and Tables

**Table 1:** Factors and levels of central composite design (*α* = 1).

Name	Units	Low	High	−*α*	+*α*
Ethanol concentration	%	80	100	80	100
Ethanol volume	L	4	6	4	6
Decoction time	Hour	0.5	3	0.5	3

*α* means the distance between the “star” point and the central point.

**Table 2:** Experiment schedules of *Salvia miltiorrhiza* alcohol extraction (*α* = 1).

Run	*A*: ethanol concentration/%	*B*: ethanol volume/L	*C*: decoction time/hour
1	90	5	1.75
2	90	5	3.00
3	80	4	0.50
4	80	4	3.00
5	90	4	1.75
6	90	6	1.75
7	100	6	0.50
8	80	6	3.00
9	100	5	1.75
10	90	5	1.75
11	100	4	0.50
12	90	5	1.75
13	80	5	1.75
14	100	4	3.00
15	100	6	3.00
16	80	6	0.50
17	90	5	1.75
18	90	5	0.50
19	90	5	1.75
20	90	5	1.75

**Table 3:** The contents of cryptotanshinone and tanshinone IIA of tanshinone extract powders.

Type	Index	Contents/mg·g^−1^	Relative standard deviation
Homemade	Cryptotanshinone	21 ± 22	1.0
(20 batches)	Tanshinone IIA	25 ± 27	1.1
Commercial	Cryptotanshinone	18 ± 18	1.0
(30 batches)	Tanshinone IIA	12 ± 28	2.3

**Table 4:** Results of physical attributes tests of tanshinone extract powders.

Type	Index	Values	Relative standard deviation
Homemade (20 batches)	Specific surface area/m^2^·g^−1^	0.240 ± 0.0663	0.276
	*D*_10_/*μ*m	12.05 ± 5.335	0.4427
	*D*_50_/*μ*m	52.52 ± 12.36	0.2353
	*D*_90_/*μ*m	126.1 ± 19.20	0.1523
	Tapped density/g·cm^−3^	0.72 ± 0.060	0.083
	Moisture/%	3.01 ± 1.22	0.405

Commercial (30 batches)	Specific surface area/m^2^·g^−1^	0.317 ± 0.0546	0.1722
	*D*_10_/*μ*m	6.917 ± 1.466	0.2119
	*D*_50_/*μ*m	27.49 ± 11.57	0.4209
	*D*_90_/*μ*m	101.0 ± 31.20	0.3089
	Tapped density/g·cm^−3^	0.73 ± 0.070	0.096
	Moisture/%	3.21 ± 0.846	0.264

The *D*_10_, *D*_50_, and *D*_90_ values indicate the maximal particle size diameter that includes 10%, 50%, and 90% of particles, respectively.

**Table 5:** The contents of cryptotanshinone and tanshinone IIA, bulk, and tapped density of five samples exemplified.

Sample	Cryptotanshinone content/mg·g^−1^	Tanshinone IIA content/mg·g^−1^	Tapped density/g·cm^−3^
1	3.4 ± 0.040	1.5 ± 0.015	0.74 ± 0.032
2	37 ± 0.036	2.5 ± 0.027	0.74 ± 0.0016
3	33 ± 0.0086	28 ± 0.047	0.74 ± 0.0013
4	4.0 ± 0.043	2.0 ± 0.034	0.70 ± 0.030
5	4.3 ± 0.015	2.9 ± 0.021	0.77 ± 0.0018

**Table 6:** Comparison of different preprocessing methods of partial least squares model for content of cryptotanshinone/mg·g^−1^.

Preprocessing method	LVs	Calibration set				Validation set			
		*r*_cal_	RMSEC	RMSECV	BIAS_cal_	*r*_pre_	RMSEP	RPD	BIAS_pre_
Raw	10	0.9922	0.0026	0.0049	0.0019	0.9798	0.0027	4.4	0.0021
S-G smooth	10	0.9921	0.0026	0.0049	0.0019	0.9796	0.0027	4.4	0.0022
Normalization	11	0.9963	0.0018	0.0033	0.0013	0.9969	0.0013	8.9	0.0011
S-T	11	0.9955	0.0020	0.0041	0.0015	0.9922	0.0014	8.1	0.0011
MSC	14	0.9983	0.0012	0.0029	0.0010	0.9921	0.0015	7.9	0.0012
S-G 1st	6	0.9883	0.0032	0.0041	0.0023	0.9949	0.0020	5.9	0.0016
S-G 2nd	6	0.9934	0.0024	0.0043	0.0016	0.9910	0.0015	7.7	0.0014
Baseline	8	0.9845	0.0036	0.0055	0.0025	0.9882	0.0021	5.6	0.0017
SNV	11	0.9962	0.0018	0.0037	0.0015	0.9963	0.0015	8.1	0.0010
WDS	8	0.9799	0.0041	0.0059	0.0029	0.9814	0.0023	5.1	0.0020

Raw means using the original spectra without any pretreatment; LVs means numbers of latent factors of the PLS model. r_cal_ and r_pre_ represent correlation coefficients for calibration and validation sets, respectively. RMSEC, RMSECV, and RMSEP represent the root mean square error of calibration, cross validation, and prediction, respectively. BIAS_cal_ and BIAS_pre_ represent bias for calibration and validation, respectively. RPD means relative predictive deviation.

S-G smooth means Savitzky-Golay smoothing; S-T represents spectroscopic transformation; MSC means multiplicative scatter correction; S-G 1st is Savitzky-Golay smoothing plus first-order derivatives for short; S-G 2nd means Savitzky-Golay smoothing plus first-order derivatives; baseline means baseline correction; SNV represents standard normal variate transformation and WDS is wavelet denoise of spectra for short.

**Table 7:** Comparison of different preprocessing methods of the partial least squares model for the content of tanshinone IIA/mg·g^−1^.

Preprocessing method	LVs	Calibration set				Validation set			
		*r*_cal_	RMSEC	RMSECV	BIAS_cal_	*r*_pre_	RMSEP	RPD	BIAS_pre_
Raw	10	0.9893	0.0043	0.0093	0.0036	0.9484	0.0060	2.0	0.0055
S-G smooth	13	0.9952	0.0029	0.0091	0.0019	0.9932	0.0044	2.7	0.0029
Normalization	10	0.9939	0.0033	0.0063	0.0027	0.9716	0.0043	2.7	0.0039
S-T	12	0.9965	0.0025	0.0066	0.0020	0.9921	0.0034	8.1	0.0022
MSC	14	0.9984	0.0017	0.0044	0.0014	0.9943	0.0024	5.0	0.0017
S-G 1st	8	0.9953	0.0029	0.0060	0.0021	0.9957	0.0019	6.2	0.0015
S-G 2nd	4	0.9915	0.0039	0.0056	0.0027	0.9955	0.0021	5.6	0.0016
Baseline	10	0.9899	0.0022	0.0055	0.0033	0.9529	0.0048	2.5	0.0041
SNV	11	0.9973	0.0018	0.0037	0.0018	0.9977	0.0015	3.6	0.0018
WDS	9	0.9772	0.0063	0.0106	0.0045	0.9532	0.0059	2.0	0.0046

**Table 8:** The best preprocessing methods for near infrared spectra of partial least squares models of physical attributes.

Index	Processing method	LVs	Calibration				Validation			
			*r*_cal_	RMSEC	RMSECV	BIAS_cal_	*r*_pre_	RMSEP	RPD	BIAS_pre_
SSA/m^2^·g^−1^	S-G 1st	11	0.9591	0.021	0.045	0.017	0.8282	0.025	1.7	0.020
*D*_10_/*μ*m	S-G 1st	12	0.9867	0.74	2.4	0.56	0.9720	0.76	2.8	0.54
*D*_50_/*μ*m	S-G 1st	5	0.9392	6.0	7.1	4.6	0.9561	4.1	3.3	3.28
*D*_90_/*μ*m	S-T	11	0.9477	10	16	7.5	0.9058	8.7	2.5	6.3
*D*_*t*_/g·cm^−3^	WDS	10	0.8830	0.034	0.038	0.027	0.8940	0.023	1.9	0.019
Moisture/%	S-G 1st	12	0.9679	0.26	0.68	0.18	0.9191	0.33	2.6	0.25

LVs means numbers of latent factors of the PLS model. r_cal_ and r_pre_ represent correlation coefficients for calibration and validation sets, respectively. RMSEC, RMSECV, and RMSEP represent the root mean square error of calibration, cross validation, and prediction, respectively. BIAS_cal_ and BIAS_pre_ represent bias for calibration and validation, respectively. RPD means relative predictive deviation.

**Table 9:** The best preprocessing methods for near infrared spectra of the least squares support vector machine model for different quality attributes.

Index	Processing method	gam	sig^2^	Calibration set		Validation set			
				*r*_cal_	BIAS_cal_	*r*_pre_	RMSEP	RPD	BIAS_pre_
Cc/mg·g^−1^	S-G 1st	2299.6	50195	0.9980	9.3 × 10^−4^	0.9985	0.0020	15	6.5 × 10^−4^
IIA/mg·g^−1^	S-G smooth	15042	5963.3	0.9996	6.9 × 10^−4^	0.9978	0.0010	12	7.2 × 10^−4^
SSA/m^2^·g^−1^	Normalization	17.4749	2541.4	0.9207	0.023	0.9661	0.017	2.5	0.016
*D*_10_/*μ*m	S-G 1st	212.18	4476.9	0.9908	0.42	0.9723	0.67	3.2	0.49
*D*_50_/*μ*m	Raw	25.830	2222.9	0.9604	3.9	0.9795	3.1	4.3	2.69
*D*_90_/*μ*m	Normalization	1928.5	2106.5	0.9835	3.8	0.9276	7.8	2.7	5.8
*D*_*t*_/g·cm^−3^	S-g smooth	6.9355	111.63	0.9851	0.011	0.8875	0.020	2.2	0.018
Moisture/%	Baseline	2042.3	1970.5	0.8900	0.28	0.9336	0.30	2.9	0.25

Gam and sig^2^ are two tuned hyperparameters of LS-SVM model. *r*_cal_ and *r*_pre_ represent correlation coefficient for calibration and validation sets, respectively. RMSEP represents the root mean square error of prediction. BIAS_cal_ and BIAS_pre_ represent bias for calibration and validation, respectively. RPD means relative predictive deviation.

Cc and IIA mean the content of cryptotanshinone and tanshinone IIA, respectively. SSA, *D*_*t*_ mean specific surface area and tapped density, respectively. The *D*_10_, *D*_50_ and *D*_90_ values represent the maximal particle size diameters that include 10%, 50% and 90% of the particles, respectively.
